# Supplementary material for: MEME-LaB: motif analysis in clusters
Source: Bioinformatics. 2013 May 14;29(13):1696–7. doi: 10.1093/bioinformatics/btt248 (PMC3694638; doi:10.1093/bioinformatics/btt248)
Supplement: Supplementary Data [file supp_btt248_Supplementary_Data.zip › Supplementary_Data/Results_Files/which_gene_ids/cluster12_motif2.html]

Cluster 12 Motif 2


Cluster 12 Motif 2 is found in the following promoters

| Gene ID | Strand | Position\* |
| --- | --- | --- |

|  |  |  |
| --- | --- | --- |
| AT1G01360 | - | 416/500 |
| AT1G01540 | + | 1/500 |
| AT1G04550 | + | 218/500 |
| AT1G08110 | + | 245/500 |
| AT1G09415 | + | 290/500 |
| AT1G09570 | - | 142/500 |
| AT1G10820 | + | 438/500 |
| AT1G12010 | - | 71/500 |
| AT1G13640 | - | 258/500 |
| AT1G15180 | + | 382/500 |
| AT1G17050 | + | 455/500 |
| AT1G17190 | + | 248/500 |
| AT1G17200 | - | 397/500 |
| AT1G18450 | - | 150/500 |
| AT1G18650 | + | 211/500 |
| AT1G19600 | + | 288/500 |
| AT1G20823 | + | 376/500 |
| AT1G22850 | - | 265/500 |
| AT1G23030 | - | 56/500 |
| AT1G24450 | + | 429/500 |
| AT1G26100 | + | 44/500 |
| AT1G28400 | + | 221/500 |
| AT1G29395 | + | 125/500 |
| AT1G29700 | - | 380/500 |
| AT1G32520 | - | 372/500 |
| AT1G34000 | - | 24/500 |
| AT1G45207 | + | 219/500 |
| AT1G45474 | + | 254/500 |
| AT1G49430 | + | 8/500 |
| AT1G49730 | + | 151/500 |
| AT1G50290 | - | 420/500 |
| AT1G51310 | + | 30/500 |
| AT1G52040 | + | 309/500 |
| AT1G52500 | + | 56/500 |
| AT1G52580 | + | 18/500 |
| AT1G53160 | - | 27/500 |
| AT1G54020 | - | 264/500 |
| AT1G56230 | - | 449/500 |
| AT1G58225 | - | 204/500 |
| AT1G63260 | - | 267/500 |
| AT1G63800 | - | 49/500 |
| AT1G64400 | - | 320/500 |
| AT1G64670 | + | 358/500 |
| AT1G65080 | - | 410/500 |
| AT1G66180 | + | 401/500 |
| AT1G67080 | + | 382/500 |
| AT1G67750 | - | 83/500 |
| AT1G68220 | + | 376/500 |
| AT1G68400 | + | 99/500 |
| AT1G68830 | + | 194/500 |
| AT1G69440 | + | 428/500 |
| AT1G69700 | + | 446/500 |
| AT1G69730 | + | 449/500 |
| AT1G70070 | - | 327/500 |
| AT1G70550 | - | 348/500 |
| AT1G70560 | - | 93/500 |
| AT1G71480 | + | 159/500 |
| AT1G72180 | + | 51/500 |
| AT1G73170 | + | 488/500 |
| AT1G74160 | + | 112/500 |
| AT1G75410 | + | 338/500 |
| AT1G75770 | + | 436/500 |
| AT1G75820 | - | 124/500 |
| AT1G78170 | - | 379/500 |
| AT1G78820 | - | 164/500 |
| AT1G80480 | - | 146/500 |
| AT2G01860 | - | 294/500 |
| AT2G01918 | + | 370/500 |
| AT2G02170 | + | 414/500 |
| AT2G02820 | - | 206/500 |
| AT2G03710 | + | 326/500 |
| AT2G04450 | + | 81/500 |
| AT2G04690 | + | 375/500 |
| AT2G05620 | + | 347/500 |
| AT2G12462 | + | 337/500 |
| AT2G13360 | + | 300/500 |
| AT2G16050 | + | 466/500 |
| AT2G16485 | - | 286/500 |
| AT2G17040 | + | 47/500 |
| AT2G17230 | + | 382/500 |
| AT2G17550 | + | 275/500 |
| AT2G18390 | - | 351/500 |
| AT2G18790 | - | 449/500 |
| AT2G19940 | + | 102/500 |
| AT2G22125 | + | 176/500 |
| AT2G22800 | - | 14/500 |
| AT2G22980 | + | 285/500 |
| AT2G23610 | - | 287/500 |
| AT2G24290 | - | 40/500 |
| AT2G25920 | + | 57/500 |
| AT2G26975 | - | 144/500 |
| AT2G28150 | - | 130/500 |
| AT2G29670 | + | 222/500 |
| AT2G31790 | - | 212/500 |
| AT2G33060 | + | 215/500 |
| AT2G33250 | - | 238/500 |
| AT2G33400 | - | 381/500 |
| AT2G33810 | + | 205/500 |
| AT2G34750 | - | 341/500 |
| AT2G35700 | + | 322/500 |
| AT2G38160 | + | 271/500 |
| AT2G38800 | + | 76/500 |
| AT2G39450 | + | 13/500 |
| AT2G41940 | + | 170/500 |
| AT2G42300 | + | 199/500 |
| AT2G43100 | - | 9/500 |
| AT2G43200 | + | 487/500 |
| AT2G43340 | - | 63/500 |
| AT2G43710 | + | 456/500 |
| AT2G44050 | - | 280/500 |
| AT2G44920 | - | 66/500 |
| AT2G45290 | + | 201/500 |
| AT2G46710 | + | 67/500 |
| AT2G46920 | + | 40/500 |
| AT2G47060 | + | 390/500 |
| AT2G47450 | + | 473/500 |
| AT2G47930 | + | 345/500 |
| AT3G01180 | - | 85/500 |
| AT3G02870 | + | 259/500 |
| AT3G04140 | - | 187/500 |
| AT3G04260 | + | 489/500 |
| AT3G05900 | + | 124/500 |
| AT3G06660 | - | 273/500 |
| AT3G06750 | + | 366/500 |
| AT3G11490 | + | 68/500 |
| AT3G11670 | - | 166/500 |
| AT3G11750 | + | 325/500 |
| AT3G15650 | - | 353/500 |
| AT3G15890 | + | 420/500 |
| AT3G17130 | + | 12/500 |
| AT3G19850 | + | 229/500 |
| AT3G20500 | + | 326/500 |
| AT3G21760 | + | 35/500 |
| AT3G21950 | - | 219/500 |
| AT3G23120 | - | 56/500 |
| AT3G23805 | + | 309/500 |
| AT3G25440 | + | 417/500 |
| AT3G26570 | + | 332/500 |
| AT3G26960 | + | 392/500 |
| AT3G27170 | + | 448/500 |
| AT3G28070 | - | 328/500 |
| AT3G30300 | - | 185/500 |
| AT3G45160 | + | 105/500 |
| AT3G46900 | + | 61/500 |
| AT3G48410 | - | 189/500 |
| AT3G48500 | + | 432/500 |
| AT3G50450 | - | 190/500 |
| AT3G51420 | + | 299/500 |
| AT3G51950 | + | 74/500 |
| AT3G52170 | - | 307/500 |
| AT3G52900 | + | 440/500 |
| AT3G54600 | - | 332/500 |
| AT3G57050 | - | 432/500 |
| AT3G57080 | - | 315/500 |
| AT3G57320 | - | 120/500 |
| AT3G58670 | - | 192/500 |
| AT3G59300 | + | 199/500 |
| AT3G61310 | - | 402/500 |
| AT3G61820 | + | 232/500 |
| AT4G00560 | - | 252/500 |
| AT4G01040 | - | 84/500 |
| AT4G01995 | - | 462/500 |
| AT4G04630 | - | 72/500 |
| AT4G04750 | - | 349/500 |
| AT4G08920 | - | 486/500 |
| AT4G09810 | - | 157/500 |
| AT4G09900 | + | 19/500 |
| AT4G09970 | + | 347/500 |
| AT4G10770 | + | 335/500 |
| AT4G11900 | - | 113/500 |
| AT4G15780 | + | 54/500 |
| AT4G15820 | - | 422/500 |
| AT4G16980 | - | 7/500 |
| AT4G17870 | + | 138/500 |
| AT4G20170 | + | 198/500 |
| AT4G22130 | + | 18/500 |
| AT4G22730 | - | 101/500 |
| AT4G23880 | + | 337/500 |
| AT4G25600 | - | 81/500 |
| AT4G25910 | + | 7/500 |
| AT4G29905 | - | 292/500 |
| AT4G31115 | - | 414/500 |
| AT4G31590 | - | 141/500 |
| AT4G31650 | + | 340/500 |
| AT4G34730 | - | 260/500 |
| AT4G36190 | - | 482/500 |
| AT4G36910 | - | 223/500 |
| AT4G37300 | - | 446/500 |
| AT4G38050 | - | 104/500 |
| AT4G38530 | - | 451/500 |
| AT4G38820 | + | 386/500 |
| AT4G38970 | - | 216/500 |
| AT5G01920 | - | 297/500 |
| AT5G03050 | - | 198/500 |
| AT5G03406 | + | 445/500 |
| AT5G04230 | - | 26/500 |
| AT5G06510 | - | 47/500 |
| AT5G07180 | + | 213/500 |
| AT5G07820 | - | 300/500 |
| AT5G07890 | - | 459/500 |
| AT5G09760 | + | 106/500 |
| AT5G10180 | + | 427/500 |
| AT5G10690 | + | 125/500 |
| AT5G13120 | - | 349/500 |
| AT5G13870 | - | 94/500 |
| AT5G14390 | - | 27/500 |
| AT5G16180 | - | 351/500 |
| AT5G16540 | + | 272/500 |
| AT5G16620 | + | 295/500 |
| AT5G17550 | + | 42/500 |
| AT5G17660 | + | 4/500 |
| AT5G18360 | - | 93/500 |
| AT5G21326 | + | 376/500 |
| AT5G21930 | + | 326/500 |
| AT5G22020 | - | 215/500 |
| AT5G22050 | - | 459/500 |
| AT5G22510 | + | 341/500 |
| AT5G23120 | + | 21/500 |
| AT5G23920 | + | 478/500 |
| AT5G25240 | - | 42/500 |
| AT5G26790 | + | 304/500 |
| AT5G27950 | + | 150/500 |
| AT5G33280 | + | 118/500 |
| AT5G37360 | + | 372/500 |
| AT5G38990 | - | 389/500 |
| AT5G39830 | - | 389/500 |
| AT5G42100 | + | 343/500 |
| AT5G42750 | + | 162/500 |
| AT5G42825 | + | 422/500 |
| AT5G43600 | + | 401/500 |
| AT5G43850 | - | 149/500 |
| AT5G45310 | + | 302/500 |
| AT5G45650 | - | 475/500 |
| AT5G45760 | - | 427/500 |
| AT5G45940 | - | 282/500 |
| AT5G46110 | + | 178/500 |
| AT5G46700 | + | 457/500 |
| AT5G47380 | + | 352/500 |
| AT5G47820 | - | 352/500 |
| AT5G48150 | + | 322/500 |
| AT5G48220 | - | 281/500 |
| AT5G48300 | + | 482/500 |
| AT5G49990 | + | 347/500 |
| AT5G51020 | - | 56/500 |
| AT5G51140 | + | 274/500 |
| AT5G53160 | - | 120/500 |
| AT5G53880 | + | 483/500 |
| AT5G54145 | + | 388/500 |
| AT5G57350 | - | 34/500 |
| AT5G59020 | - | 52/500 |
| AT5G59250 | + | 382/500 |
| AT5G60840 | + | 222/500 |
| AT5G62140 | + | 466/500 |
| AT5G63500 | + | 199/500 |
| AT5G64280 | + | 340/500 |
| AT5G66490 | - | 197/500 |
| AT5G67160 | - | 217/500 |
| AT5G67385 | - | 420/500 |

**\*** Overlapping sequences are merged to avoid redundancy. As a result some sequences in the final set may be longer than the specified maximum.

Distribution

+

-

Position values represent number of bases from the 5 prime end of the sequence provided to MEME and the length of that sequence. It is these figures expressed as proportions that are used for statistical analysis.

Probability value of comparison of these values to a linear distribution, p = 0.47

The motif is found 143 times on the + stand and 115 times on the - strand.

Probability value that it is evenly distributed between the two strands, p = 0.046

Move the mouse pointer over the gene ids to highlight the corresponding marker
